# Approach of Pavement Surface Layer Degradation Caused by Tire Contact Using Semi-Analytical Model

**DOI:** 10.3390/ma14092117

**Published:** 2021-04-22

**Authors:** Edem Yawo Manyo, Benoit Picoux, Philippe Reynaud, Rémi Tautou, Daniel Nelias, Fatima Allou, Christophe Petit

**Affiliations:** 1 Laboratoire GC2D, EA 3178, University of Limoges, Bd J. Derche, F-19300 Egletons, France; edem-yawo.manyo@unilim.fr (E.Y.M.); benoit.picoux@unilim.fr (B.P.); philippe.reynaud@unilim.fr (P.R.); remi.tautou@unilim.fr (R.T.); fatima.allou@unilim.fr (F.A.); 2 INSA Lyon, University of Lyon, LAMCOS, CNRS UMR5259, Avenue J. Capelle, F-69100 Villeurbanne, France; daniel.nelias@insa-lyon.fr

**Keywords:** tire-pavement contact, rolling contact, rubbing, semi-analytical method, top-down cracking, kalker’s theory

## Abstract

New methods of degradations on the pavement’s surface, such as top-down cracking and delamination, caused by the repeated passage of heavy vehicles led to questions about the impact of the contact between the tire and the pavement. In fact, to increase the service life of the structures, future road design methods must have a precise knowledge of the consequences of the contact parameters on the state of stress and deformation in the pavement. In this paper, tractive rolling contact under the effect of friction is modeled by Kalker’s theory using a semi-analytical method (SAM). A tire profile is performed thanks to a digitization by fringes or a photogrammetry technique. The effect of rolling on the main surface extension deformations is then highlighted to study top cracking. At the end of the SAM calculation, contact areas are closed to 200 μdef, exceeding the allowable micro-deformation limit for the initiation of cracking. In addition, results on the main strain directions also give information on the direction of cracking (initiation of longitudinal or transverse cracks). The cracking then becomes evident, leading to a reduced service life.

## 1. Introduction

The road sector has grown considerably since the middle of the last century. The increase in traffic and the level of loading, combined with the aggressiveness of the tires and the large amplitudes of climatic variation, have led to an increase in the deterioration of the roads and, consequently, to an increase in pavement maintenance. Bottom cracking and decohesion at the interfaces are degradation problems already well known by road companies. Recently, new types of degradations, named Top-Down Cracking (TDC), have appeared. These degradations are initiated at the surface and in the upper layer of the pavement. Many studies on the generation and propagation of these degradations currently use a uniformly distributed loading to model the action of the tire on the surface without considering the geometry and the real distribution of the contact pressures. This very simplistic assumption is fully accepted in regard to the prediction of the service life of the deep layers of pavements. It is well known that unfavorable “sizing” degradations come up from the base of structures by traction (cracking) or rutting of the support soil. However, when one is interested in the degradations coming from the surface, the tire–road contact must be well modeled since, in any case, the distribution of pressure is uniform.

Researchers have tried to explain the initiation of the cracks at the surface of pavement (Asphalt Concrete layer). Many recent studies have shown the TDC mechanism of pavements. Cracking can begin on the surface of the pavement and spread downward (downward cracking). Several factors affect TDC [1]: load (tensile, shear), material (low breaking energies, aging), construction (joint, segregation) and temperature (thermal stress), but the shear stress generated by tire tension on the pavement surface is the most important factor in initiating the surface cracking mechanism. The tensile and shear stress generated by repeated loading triggers the material damage process, while the thermal stress during daily thermal cycles increases damage evolution. Tamagny [2] showed that the viscoelasticity of pavement can explain the top-down cracking caused by the traction zone behind the tire.

The development of the TDC was reported as a very important problem over the past decade. Hugo [3] observed the initiation of road surface cracks under the tire pressure area of the truck simulator and noted that the cracks were caused by horizontal forces induced on the contact surface between the tire and the roadway. Centripetal radial stresses were cited as inducing the surface tension of the pavement at the edge of a circularly distributed load by Kunst [4]. The latter also proposed a model based on distortion energy to best explain the development of surface-initiated cracking. It has also been reported that the calculated distortion energy is always more critical in the sub-layer rather than the surface of the asphalt layer. Matsuno [5] reported that longitudinal surface cracks are due to large extensional strains generated near the edges of tires at elevated temperatures. They explained that extension strains are often concentrated in the vicinity of an induced small crack on the surface of the roadway. However, the analysis work was limited to a traditional uniform circular vertical loading. An analysis of the cause of micro-mechanical downward cracking was conducted by Wang [6]. The authors found that secondary tensile stress could be induced by shear loading due to expansion. They suggested that the overall skeletal structure of particles and the concentration of mastics are two important factors that can affect downward cracks. Wang [7] found, using the viscoelastic border element method, that the residual viscoelastic stress due to the load may be another potential mechanism for top-down cracking. The works of Yoo [8] have shown that vertical shear strain at the edge of the tire is more critical than extensional strain. Petit [9] has shown that the TDC fatigue of a pavement structure is initiated by superficial extension strains that are induced by tangential stresses applied to the surface of the roadway. A crack initiation model was developed by Lytton [10]. The authors found that the number of charge cycles to achieve failure could be predicted with good accuracy by considering the original stiffness, the stress state expressed in terms of average principal stress, the octahedral shear stress at the bottom of the layer, the percentage of air void and the bituminous material in the mixture. A model of “Von Mises strain” was proposed by Sousa [11] to simultaneously consider normal and shear strain. The fatigue resistance of the bending fatigue test with strain control is thus calculated. After the laboratory tests, Kunst [4] argues that only a combination of large surface dissipated energy, shear stresses, temperature-induced stresses and residual stresses could cause surface cracking of the roadway. In a “Laboratoire Central des Ponts et Chaussées” report [12], the laboratory tests show that the rate of evolution of the TDC to factors that favor the aging of the bituminous mix (high temperatures, high voids and binder deficiency), the contraction and fatigue (low temperature, high daily temperature range). According to a study conducted by Roque [13], downward cracking could be caused by surface traction induced by bending from the tire into low or medium thickness bituminous layers, or near shear-induced surface traction at the edge of the tire-thick bituminous layers. In their study, a continuous viscoelastic damage model and a fracture mechanics model was used to predict the initiation and propagation of the crack. However, the developed model is still not suitable for integrating and developing a model of top-down crack performance prediction for empirical-mechanical design. The impact of the tire’s treads and its aggressiveness on the road surface has already been demonstrated by Reynaud [14]. The authors developed a precise modeling of the contact using a semi-analytical tribological approach under stationary conditions. This approach, more detailed in parts 2 and 3, was used for several years in the modeling of mechanical contact (ball bearing, for example). The recent adaptation of this approach to tire-pavement contact is, on the one hand, different from other existing semi-analytical model and, on the other hand, a challenge considering the reality of the tire geometry and the complexity of the pavement structure combining the speed of calculation. They concluded that a more precise knowledge of the tire-pavement contact represents an important way to finely describe the surface degradations. With this knowledge, it will be possible to envisage the construction of a rational method for pavement maintenance.

In this paper, we approach the tractive rolling contact, defined by the rolling between two bodies transmitting tangential forces in the presence of friction. The partial slip phenomenon is then studied in stationary rolling. In this way, contact pressure, shear at the pavement surface and TDC is studied.

## 2. Formulation of the Contact Problem

The problem of contact was studied by Ziefle [15] in a rolling contact study under stationary conditions. The authors used the ALE method (Arbitrary Langrangian–Eulerian) coupled with the finite element method (FEM). In their model, they were able to show the distribution of normal, longitudinal and transverse surface stresses. This approach was also used by Wollny [16] to study the tire–road interaction, taking into account the effect of friction on the pavement and the tire. A 3D FEM model was developed by Wang [17] to study rolling contact with braking and cornering conditions. Blab [18] used contact measurements from an experimental study named SIM [19] to test a 3D FEM model and studied top-down cracking and rutting phenomena. The problem of partial slip and stationary rolling contact was taken up by Carter [20] in a 2D model and applied to the wheel-rail contact in a realistic approach. Haine [21], then Kalker [22] later developed and transformed Carter’s theory to study the problem of slip/stick and strip theory of the pavement contact area. Kalker’s linear elasticity theory is nevertheless the first used in 3D, in a semi-analytical numerical code CONTACT, allowing an accurate study of the rail–wheel contact. Throughout this paper, pressure results are presented as normalized by the Hertzian pressure. A definition of the Hertzian pressure and some theoretical details of this formulation have already been published by the authors and can be found in a previous paper [23].

## 3. The Semi-Analytical Method (SAM)

**Figure 1:**
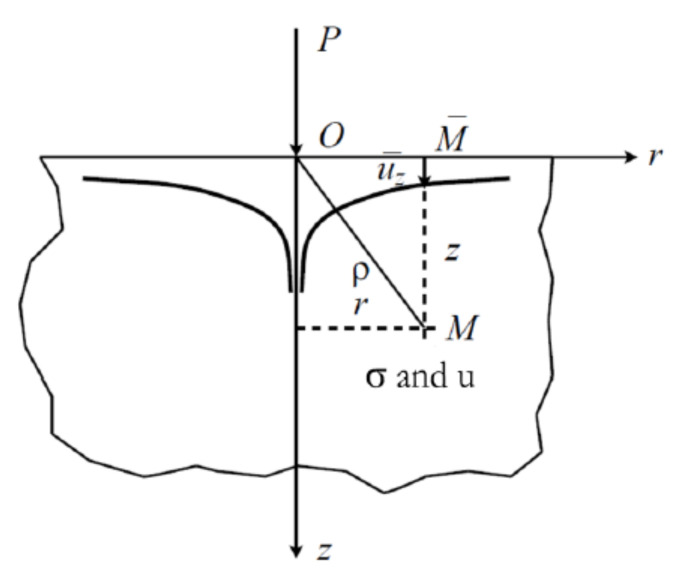
Description of the problem.

As for pavement design methods, homogeneous elastic materials can be easily accepted and are checked as Boussinesq’s solution for semi-infinite soils, and then Burminster’s model for multilayered structure. Semi-analytical methods are methods that were used many times in multilayered or contact problems. Unlike models using finite elements, these methods are simpler to use and are especially less consumer in terms of computation time. In a first approach, Reynaud [24] used this type of modeling in a calculation code named SAM to obtain the contact pressure at the surface during a tire contact. The tire and the pavement were defined as a smooth surface. The results compared to experimental results made it possible to define the contact areas and the stresses on the pavement surface. The semi-analytical method is based on the theory of semi-infinite elastic spaces. This method is the origin of the Hertzian contact. Let us suppose that an elastic half-space limited by the plane z = 0 where the boundary conditions at infinity in terms of displacement are null (see Figure 1).

The objective is to solve the problem of Newman, then to determine the elastic state of this half-space under the action of normal p(x, y) and tangential qx(x, y) and qy(x, y) stresses applied on a closed surface close to the origin. If we consider any point of coordinates (ξ,η) of the surface under loading, and any point of coordinates (x,y,z) of the half-space, we can define the distance between these two points by:

(1) $$\rho ={\left\{{\left(\xi -x\right)}^{2}+{\left(\eta -y\right)}^{2}+z^{2}\right\}}^{1/2}$$

According to Love [25], the elastic displacement components at a point M(x,y,z) in the volume of body 2 are given by:

(2) $$u_{x}=\frac{1}{4\pi G}\left\{2\frac{\partial \Gamma }{\partial z}-\frac{\partial H}{\partial x}+2\nu \frac{\partial \psi_{1}}{\partial x}-z\frac{\partial \psi }{\partial x}\right\}$$

(3) $$u_{y}=\frac{1}{4\pi G}\left\{2\frac{\partial \phi }{\partial z}-\frac{\partial H}{\partial y}+2\nu \frac{\partial \psi_{1}}{\partial y}-z\frac{\partial \psi }{\partial y}\right\}$$

(4) $$u_{z}=\frac{1}{4\pi G}\left\{\frac{\partial \Gamma }{\partial z}-\left(1-2\nu \right)\psi -z\frac{\partial \psi }{\partial z}\right\}$$

where G is the elastic shear modulus of the semi-infinite layer; Γ, H, ϕ, are potential functions related to the surface normal and shear stresses loaded. In the previous equations:

(5) $$\psi =\frac{\partial \Gamma }{\partial x}+\frac{\partial \phi }{\partial y}+\frac{\partial H}{\partial z}$$

(6) $$\psi_{1}=\int \psi \mathrm{dz}$$

The SAM code is already validated in normal elastoplastic contact between a sphere and plane by Jacq [26], both by comparison to Finite Element Analysis (FEA) and to experiments (i.e., ceramic (elastic) spheres rolling over a steel (elasto-plastic) flat). The model was also validated in the visco-elastic regime by Koumi [27,28] when a sphere is indenting, rolling or sliding against a visco-elastic half-space containing, or not, an ellipsoidal inclusion, again by comparison to FEA. A comparison is made by Nélias [29] with a finite element results model from the literature for the elasto-plastic case. A very good agreement is found when the plastic deformation is less than 5%. It relates the contact pressure distribution and contact area to the normal force or displacement. To start a computation, an upstream of the SAM characteristics is needed, such as mechanical characteristics of the bodies in contact: Young’s modulus and Poisson’s ratios, isotropic or anisotropic, homogeneity or heterogeneity and geometry of the bodies in contact. Due to the complex behavior of the tire, a simple global Young’s modulus was chosen (see Section 4). Note that our tire model is limited to a linear viscoelastic behavior—here with a simple Maxwell model—but it is possible to integrate the SAM with a complex behavior of the viscoelastic response by a series decomposition of Prony, which implies to know the response of the material corresponding to different relaxation times. For nonlinear behavior, there are several families: plasticity or damage (which reflects an irreversible phenomenon), or large displacements and large strains. In the present study and at the contact level, strains remain small.

## 4. Rolling Contact

**Figure 2:**
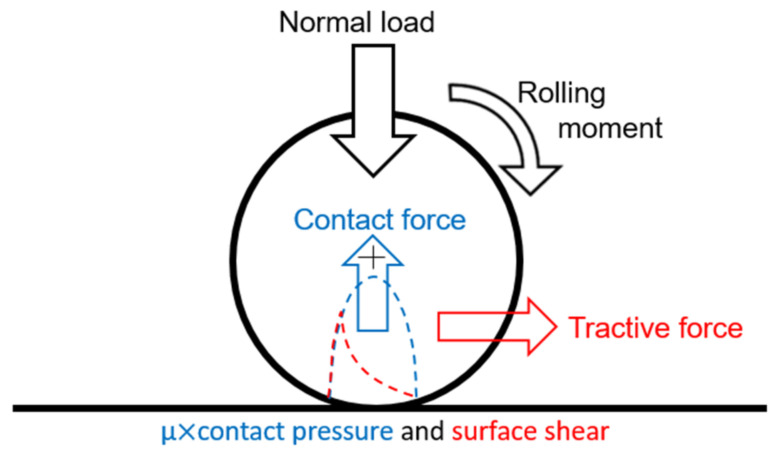
Rolling contact kinematics.

The kinematics of stationary rolling contact are presented in Figure 2.

**Figure 3:**
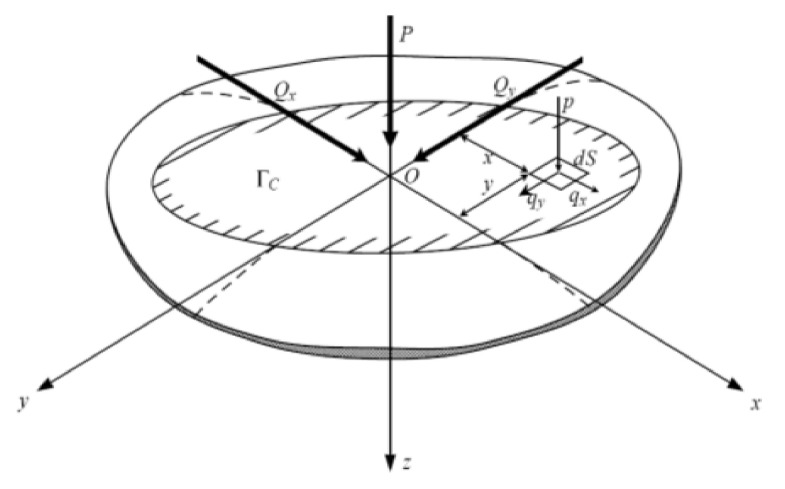
Contact forces.

For this rolling contact problem, the formation of the contact is described by the normal problem. The rolling aspect is described by a tangential problem, which considers a tangential applied force and the friction law of Coulomb. The resolution of these two problems successively allows having the surface stress fields, including the distribution of the normal and tangential stress. Figure 3 shows the forces transmitted in the contact area.

The normal problem is described by three main formulations defined by:

(7) $$\mathrm{Load}\;\mathrm{balance}:\;P=\int_{\Gamma c}p\left(x,y\right)dS$$

where P is the normal load applied, p(x, y) the contact pressure at a point (x, y) and Γc the contact area. The separation of the bodies:

(8) $$h\left(x,y\right)=h_{i}\left(x,y\right)+\delta_{z}+u_{z}^{p}\left(x,y\right)$$

where h(x, y), h_i_(x, y) are, respectively, the total and initial separations of the bodies at a point (x, y), δ_z_ the normal displacement of the rigid body and $u_{z}^{p}\left(x,y\right)$ the displacement normal elastic of both bodies due to contact pressure. The contact conditions are:

(9) $$h\left(x,y\right)=0\;\mathrm{and}\;p\left(x,y\right)>0$$

To these conditions, the condition of inter-penetration is added:

(10) $$\mathrm{if}\;h\left(x,y\right)>0\;\mathrm{then}\;p\left(x,y\right)=0$$

These equations are solved simultaneously by the discrete convolution of the fast Fourier transform (DC-FFT) and the Conjugate Gradient Method (CGM). As described in the introduction, the tangential problem is modeled by Kalker’s theory. The latter is based on a mathematical formulation of the slip equation. In stationary regime, this equation is in the form:

(11) $${\dot{s}}_{\tau }\left(x,y\right)=w_{\tau }-V\times (\partial u_{\tau }\left(x,y)/\partial x\right)$$

where ${\dot{s}}_{\tau }\left(x,y\right)$ is the slip velocity at the point (x, y), w_τ_ the rigid slip defined by the ratio of the difference between the speed of rotation and the advance of the speed of rotation, V the rolling speed, and u_τ_(x,y) the elastic tangential displacement of the two bodies (τ = x, y). The Coulomb law is defined by:

(12) $$\left|\left.q_{\tau }(x,y\right)\right|<µ\times p\left(x,y\right)\Rightarrow \left(x,y\right)\in \Gamma_{a}$$

(13) $$\left|\left.q_{\tau }(x,y\right)\right|=µ\times p\left(x,y\right)\Rightarrow \left(x,y\right)\in \Gamma_{g}$$

where q_τ_ (x, y) is the surface shear at the point (x, y), μ the coefficient of friction and Γ_a_, Γ_g_ are, respectively, the stick and sliding area of the contact surface (Γ_a_ ⋃ Γ_g_ = Γ_c_). As in the formulation of the normal problem, there is the verification of the loads’ balance:

(14) $$Q_{\tau }=\underset{\Gamma_{c}}{\int }q_{\tau }\left(x,y\right)dS$$

The resolution of these equations also uses the techniques of the DC-FFT as well as the CGM. The knowledge of the surface state stress and the equations of Love will lead to determining the stresses in the under layer. In our model, the pavement is assumed to be homogeneous elastic and modeled by a semi-infinite medium composed of asphalt concrete (AC). The tire is the body designated by the index 1 and the pavement is the body designated by the index 2. The simulation parameters are summarized in Table 1.

## 5. First Results: Contact Pressure

**Figure 4:**
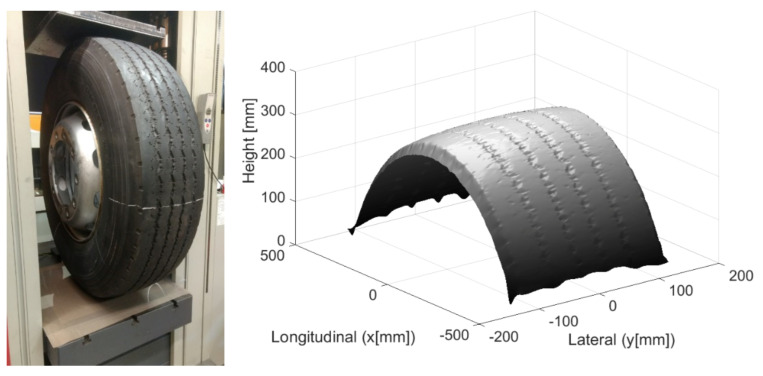
Experimental set-up and numerical reconstruction of the surface geometry.

The first results prove that tire geometry has a great influence on the contact pressures. In order to better approach this geometry, a photogrammetry method was chosen to carry out the reconstruction of the tire surface. A Michelin (315/80R22.5) (Clermont Ferrand, France) tire is thus photographed with a good resolution camera. All pictures are digitally assembled using a software, allowing a recovery of more than 80% on each picture (Figure 4). A 3D image of the tire is then obtained under its actual conditions of use (at the average inflation pressure of 8.2 bars). Using the SAM code with these results, contact pressures can be calculated. A cubic mesh (3 mm × 3 mm × 3 mm) is chosen according to sensor precision [30]. This mesh allows the calculation time to be optimized. A study of the contact pressure with the help of a tribological approach was carried out by Manyo [31].

This study used a smooth tire profile and compared numerical results to measurements from the literature. Calculations were made with SAM model. It performed with a scanned tire profile and compared to measurements made by a TekScan resistive sensor. Since the tire is not a simple mechanical system, it is not so obvious to do a simple tensile or compression test and choose a simple law of materials mechanics like Hooke’s law or others. Therefore, in order to take into account the overall behavior of the tire, it was thought that a test will highlight the tire-pavement contact aspect and choose an appropriate mechanical law. The tire is assumed to be homogeneous elastic and modeled by an equivalent Young’s modulus. Tire load-deflection curves from experimental measurements were used to calibrate the tire model parameters.

The analytical relationship is found as follows [31]:

(15) $$E_{wheel}\left(MPa\right)=2.7\times 10^{-3}P+7.0\times 10^{-1}$$

where E_wheel_ is the Young’s modulus of the tire and P the inflation pressure in kPa.

**Figure 5:**
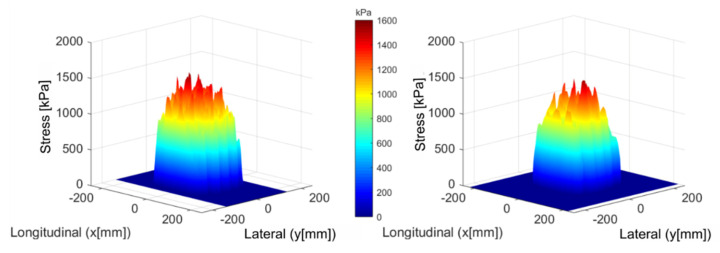
Contact pressure: SAM simulation (**left**) and TekScan measurement (**right**).

Figure 5 shows the distribution of the contact pressure field for a normal static load of 35 kN (inflation pressure is equal to 820 kPa). A good relationship between the numerical and experimental results is then observed. The area of the contact surfaces of the two results, as well as the maximum values of the contact pressure, are almost equivalent (58,761 mm^2^ for Tekscan and 59,553 mm^2^ for SAM, and 1532 kPa for Tekscan and 1576 kPa for SAM).

## 6. Results for Free Rolling

**Figure 6:**
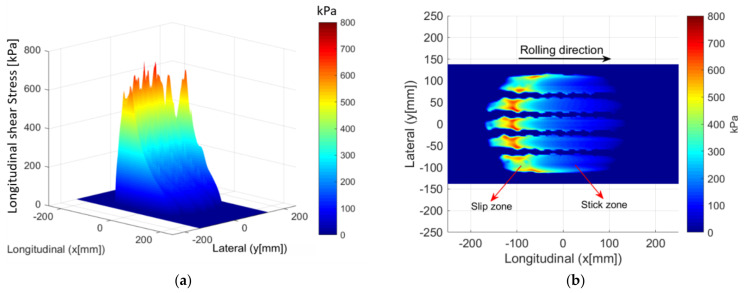
Distribution of the surface shear field stress. 3D profile (**a**); projected view highlighting the stick/slip zones (**b**).

Figure 6 shows the surface shear field for a 3D and 2D profile when a tangential force is applied in the presence of friction whose coefficient μ is 0.7. On the 2D profile, a sliding zone where there is a high concentration of stress and a zone of stick on the contact surface can be distinguished. The experimental comparison is not yet possible, but the stress profiles give us a confirmation of the consistency of the results. Similar results have indeed been found in the field of contact mechanics [22].

**Figure 7:**
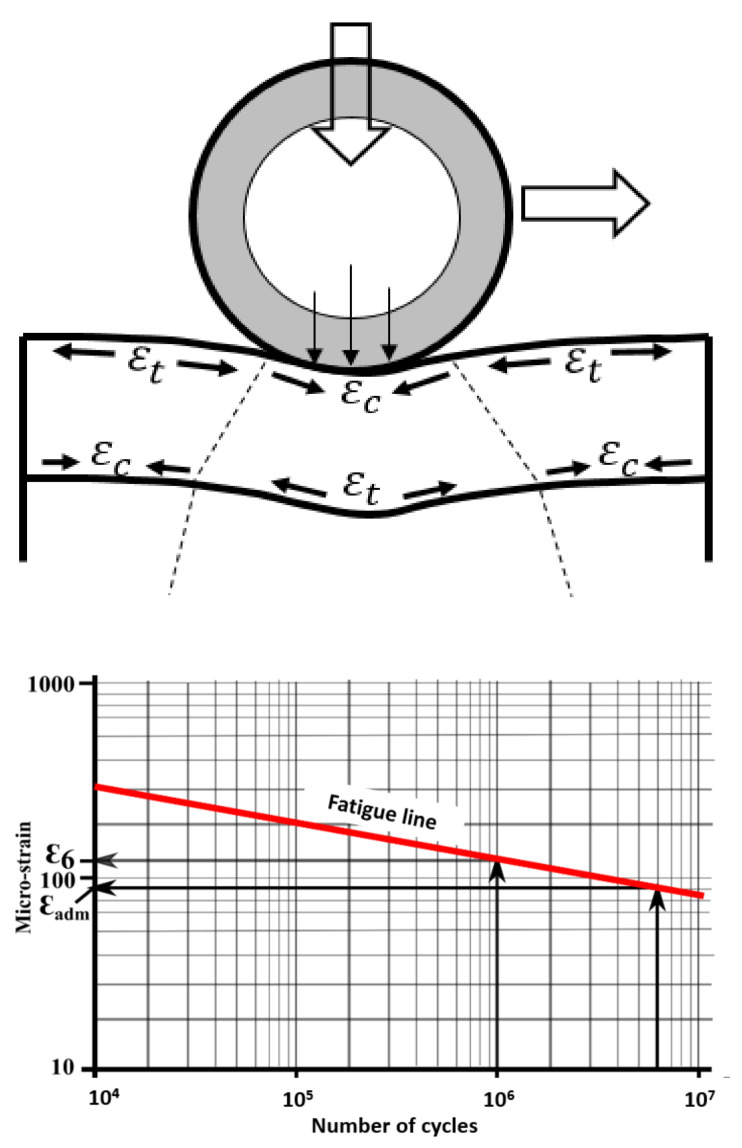
Strains present in the layers of the pavement under loading and fatigue line of bituminous mixes [32].

In fatigue modeling, it is assumed that the main strain by extension, according to the criterion of damage by Mazars [32], cracking at the bottom of the layers is at the origin of the rising or falling cracks near the surface (Figure 7). As in Pavement design methods, fatigue is assumed at the maximum strain level, considering different material and loading variabilities. These strains are often generated by the passage of heavy vehicles or the poor development of the lower layers. In the French method of pavement design, the cracks are modeled mainly as coming from the bottom of the layers. The fatigue lines of bituminous mixes presented in Figure 7 makes it possible to define their fatigue strength. ε_6_ is chosen as the critical micro-strain beyond which cracking is imminent at a low number of loading cycles (around 120 micro-strain for 10^6^ cycles). According to the design method, the strain by extension is calculated at the bottom of the layer and compared to the admissible strain (ε_adm_ close to 90 micro-strain), where it must be less than this strain in order to have a satisfactory life. Although this last criterion considers that the location of the contact is at the same place on the pavement, our model will nevertheless make it possible to properly estimate the stress levels in the first layers and to approach the different directions of cracking under different loading and friction conditions.

**Figure 8:**
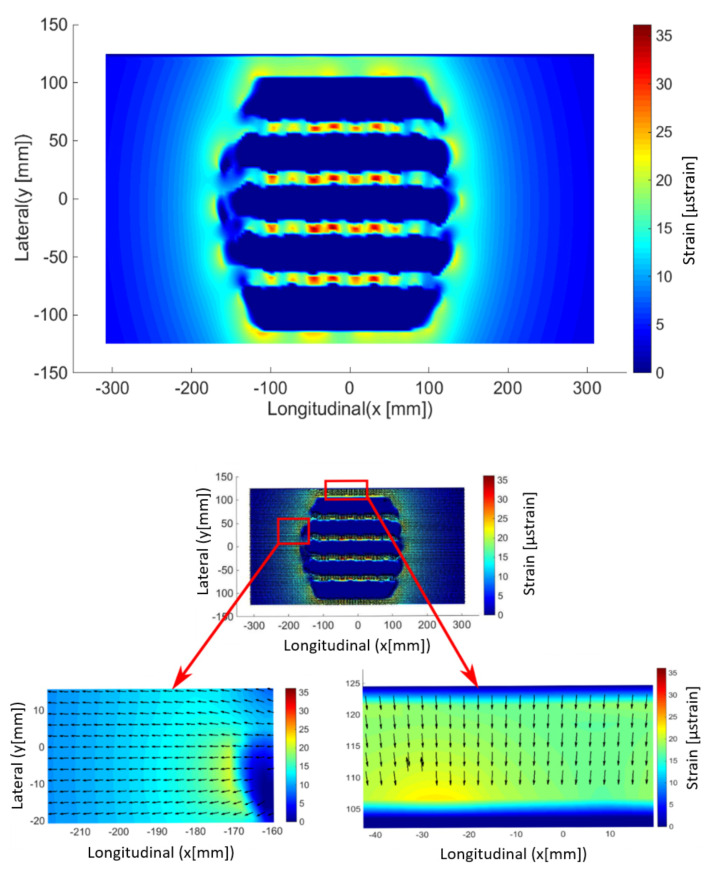
Maximum principal strain (by extension) at the pavement surface (**up**) and corresponding principal direction (**bottom**) for free rolling.

The objective of our modeling is to evaluate the effect of the tire on the surface and in the underlayer of the pavement in the presence of friction, particularly in the case of descending cracks. In the literature, the effect of the horizontal force only concern the surface layer where cracking begins and develops; this observation is given by Hammoum [33]. Therefore, the fields of maximum surface strain are presented in this section as well as the main directions. Figure 8 shows the principal strains by surface extension (z = 0) for the case of free rolling. The latter is modeled by a friction coefficient equal to 0.7 with no additional tangential load. It is observed that the peaks of high strains are between the tire grooves, which can also explain the cracking in the contact itself. On the other hand, we note that the maximum micro-strain (µdef) is around less than ε_6_. Figure 8 also shows the main directions of extension.

## 7. Results for Tractive Rolling

**Figure 9:**
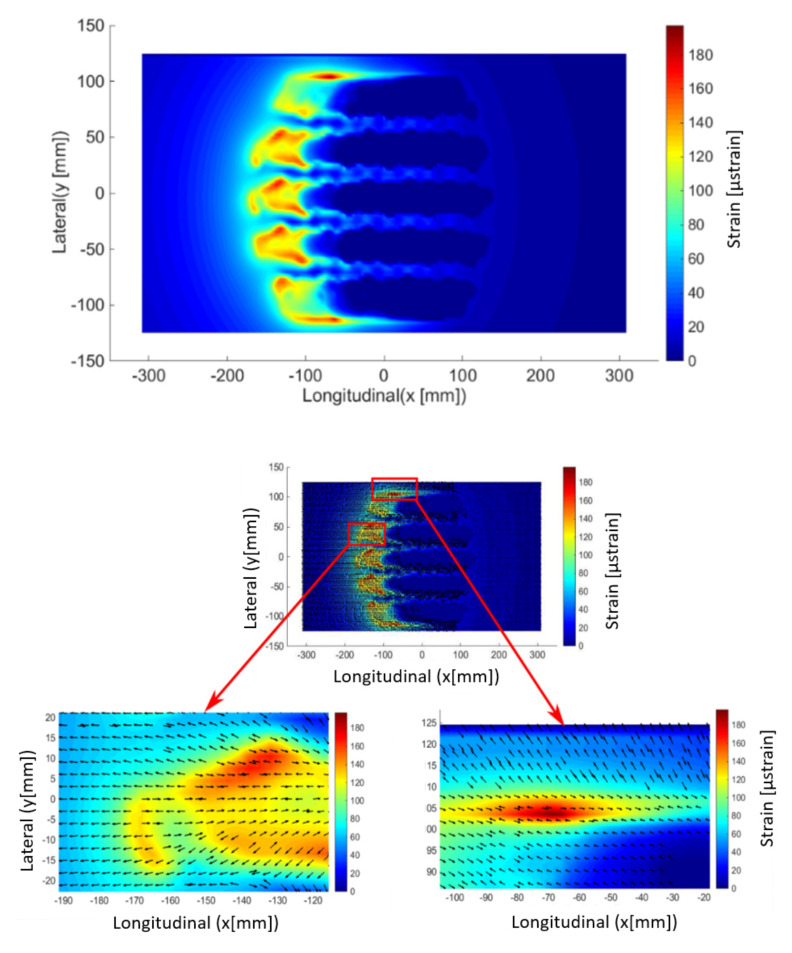
Maximum principal strain (by extension) at the pavement surface (**up**) and corresponding principal direction (**bottom**) for a tractive rolling contact.

**Figure 10:**
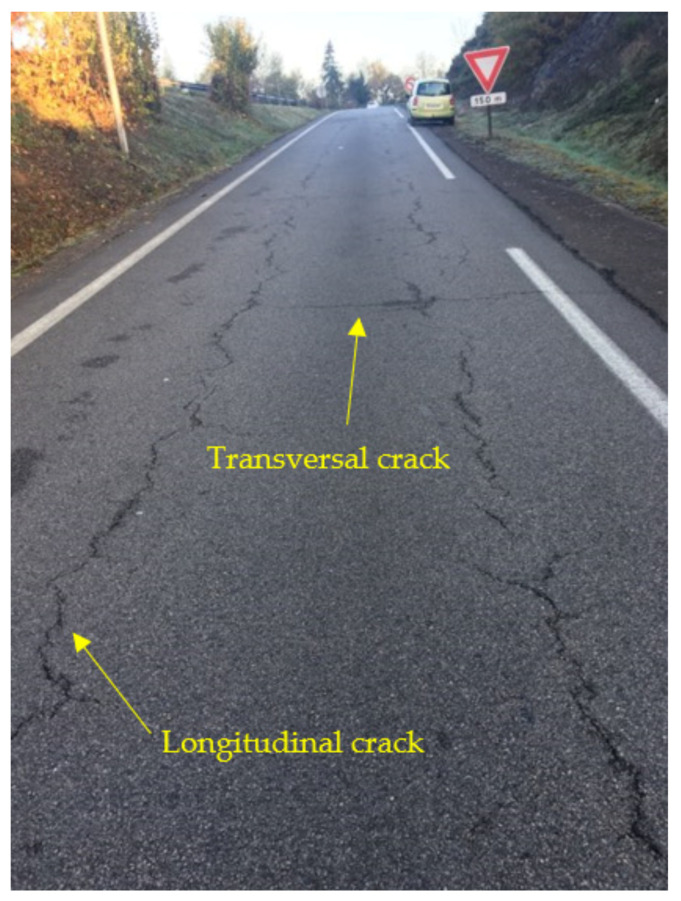
Cracking at the pavement surface.

Figure 9 shows the main surface strain field for tractive rolling (described above by Kalker’s theory). The friction coefficient between the tire and the pavement μ = 0.7 and tractive force Qx = 10 kN (see Table 1). The effect of the sliding zone (tractive rolling) on the pavement degradations is clearly observed. Note that it is in this sliding zone that the peaks of micro-strain are higher and more precisely on the edges of the contact and are of the order of 200, which is almost twice that of ε_6_. In this case, the cracking is then obvious for a very short life. This model also gives us information on the cracks in any direction. Figure 9 shows the main directions of extension in the case of the tractive bearing. In this case, some directions are almost oblique. As can be seen on pavements (for example see Figure 10), longitudinal as well as transverse cracks appear. Then, in principle, we will note that the directions orthogonal to the direction of the bearing are at the origin of the longitudinal cracks, and those parallel to the direction of the bearing are at the origin of the transverse cracks.

## 8. Conclusions

This study presented the modelling and the validation of a Semi-Analytical code for tire-pavement rolling contact analysis, allowing a more realistic, precise and, above all, faster study than the current methods, especially the Finite Element Method (FEM).

This model thus makes it possible:

- To obtain the surface shear field in the presence of friction, which is necessary during sizing and pavement damage calculations;
- To have a faster knowledge of the tensile strains at the origin of the descending cracks and the values of the maximum equivalent stresses in the under layer with considering this same friction;
- To show that the actual local pressure of the tire near grooves is actually higher than that used by conventional design models (uniform pressure), proven here by numerical study and its experimental validation;
- To allow a faster and more precise knowledge of the strains by extension at the origin of the premature appearance of descending cracks on the surface of pavements.

Finally, numerical results of strains are approximately equal to 35 µstrain maximum between tire treads during the free-rolling and 200 µstrain maximum at the edges during the tractive rolling contact. This is never observed by the models of the uniform load distribution hypothesis. In addition, results on the main strain directions also give us information on the direction of cracking (initiation of longitudinal or transverse cracks).

Subsequently, we plan to integrate transversal efforts to study tearing phenomena in singular road points (turns, roundabouts). Of course, our model does not yet take into account certain physical phenomena. Indeed, the bituminous materials have a complex behavior including both a reversible and irreversible effect. The reversible phenomenon leads to an elastic and viscoelastic behavior. For the study of cracking, a viscoelastic model will also be more suitable to understand delamination problems between the wearing course and the base layer. The pavement here is also supposed to be a monolayer, while it is a multilayer structure. Therefore, a future consideration of a multilayer model is necessary to analyze the effect of each layer on the mechanical response of the structure. Any consideration of the heterogeneities may allow having an overall mechanical behavior of the pavement.

## Figures and Tables

**Table 1:** Tire and pavement Parameters.

Parameter	Value
Normal load, P (kN)	32.5
Longitudinal tangential load, $Q_{x}$(kN)	10
Tire Young’s Modulus, $E_{1}$(MPa)	3
Tire Inflation Pressure, (bars)	8.2
Tire Poisson’s ratio, $\nu_{1}$	0.5
AC Young’s modulus, $E_{2}$(MPa)	5400
AC Poisson’s ratio, $\nu_{2}$	0.35
Friction coefficient, µ	0.7

## Data Availability

Some or all data, models, or code generated or used during the study are proprietary or confidential in nature and may only be provided with restrictions.
